# Assessment of left ventricular apical rotation in obese by Cardiovascular MR tagging

**DOI:** 10.1186/1532-429X-13-S1-P326

**Published:** 2011-02-02

**Authors:** Gabriella M Vincenti, Jean-Noel Hyacinthe, Joost PA Kuijer, Francois Mach, Osman Ratib, Thomas Schindler, Jean-Paul Vallée

**Affiliations:** 1 Division of Cardiology, Geneva University Hospital, Geneva, Switzerland; 2 Division of Radiology, Geneva University Hospital, Geneva, Switzerland; 3 Department of Physics & Medical Technology, VU University Medical Center, Amsterdam, Netherlands; 4 Division of Nuclear Medecine, Geneva University Hospital, Geneva, Switzerland

## Background

Obesity is associated with cardiac remodelling with increased left ventricular mass and end-diastolic volume. CMR is a unique technique to evaluate cardiac morphology and function in obese individuals. Among CMR techniques, myocardial tagging allows to quantify myocardial strain and left ventricular rotation along the RR cycle (1). The aim of this preliminary study was to explore the feasibility of left ventricular apical rotation quantification by CMR tagging in obese individuals and exploring possible alteration of myocardial motion pattern.

## Methods

8 healthy controls (18<BMI<25) were imaged using a 3T trio system and 7 obese subjects (BMI>30) without other cardiovascular risk factors and with normal left ventricular ejection fraction were imaged using a 1.5T Espree system (wider bore, well adapted to obese subjects). We used an ECG gated, single breath hold balanced SSFP line tagging sequence with LISA, with CSPAMM acquisition scheme (2). The field of view was 340 x340 mm, matrix 256 with 32% phase resolution, bandwidth 850 Hz, slice thickness 7 mm, tag spacing 7 mm, TE 1.28 (TE 1.54 @ 3T) and Flip angle 20° (15° @ 3T). Images were analyzed and apical rotations were computed using Extrema Temporal Chaining (ETC) (3).

## Results

**Figure 1:**
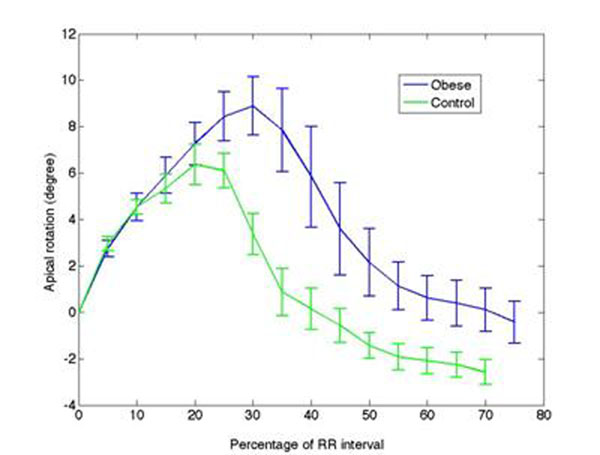
Left ventricular apical rotation along RR interval in Obese and control subjects.

The left ventricular apical systolic rotation measured by CMR tagging was increased and delayed in obese patients while the diastolic apical untwisting was prolonged, when compared to control subjects (Fig.1).

## Conclusions

Our preliminary results confirm the presence of an abnormal pattern of left ventricular rotation in obese individuals that can be identified with CMR tagging. This abnormal pattern may reflect the remodeling secondary to increased hemodynamic requirements (volume-overload and sympathetic system activation) in obese subjects, while the delayed diastolic untwisting may precede a diastolic dysfunction. These preliminary data confirm that CMR with myocardial tagging may allow identifying early abnormalities of left ventricular function in obese individuals with normal left ventricular ejection fraction.
